# Biological Characteristics, Artificial Domestication Conditions Optimization, and Bioactive Components of *Beauveria caledonica*

**DOI:** 10.3390/microorganisms12081554

**Published:** 2024-07-29

**Authors:** Wang Cao, Changxia Yu, Yan Zhao, Qunying Lin, Chunying Deng, Chuanhua Li

**Affiliations:** 1 Institute of Edible Fungi, Shanghai Academy of Agricultural Sciences, Shanghai 201403, China; caoranbiology@163.com (W.C.); ycx41529@163.com (C.Y.); 2 Nanjing Institute for Comprehensive Utilization of Wild Plants, China Co-ops, Nanjing 210110, China; linqunying1007@126.com; 3 Guizhou Institute of Biology, Guizhou Academy of Sciences, Guiyang 550001, China; mary_chunying@sina.com

**Keywords:** *Cordyceps* s.l., optimum medium, biological efficiency of fruiting bodies, polysaccharide content, adenosine content

## Abstract

In this study, one strain of *Beauveria caledonica* was isolated from wild fruiting bodies collected from Guizhou Province, China, and its species identification, biological characteristics, domestication, and cultivation methods were studied along with polysaccharide and adenosine content analysis. The mycelia were identified by ITS sequencing, and the fruiting bodies of *B. caledonica* were domestically cultivated for the first time using wheat and rice as basic cultivation media. The carbon sources, nitrogen sources, cultivation temperatures, and pH for mycelial growth were optimized through single-factor experiments and response surface methodology (RSM) experiments. The polysaccharide content was detected by the phenol–sulfuric acid method, and the adenosine content was measured by high-performance liquid chromatography (HPLC). The results confirmed that the identified mycelia were *B. caledonica*. The optimum medium for solid culture was 25.8 g/L glycerol, 10.9 g/L yeast extract, 1 g/L MgSO_4_·7H_2_O, 1 g/L KH_2_PO_4_, 10 mg/L vitamin B_1_, and 20 g/L agar; the optimum pH was 6.5, and the optimum culture temperature was 25 °C. The optimal liquid culture medium was 26.2 g/L glycerol, 11.1 g/L yeast extract, 1 g/L MgSO_4_·7H_2_O, 1 g/L KH_2_PO_4_, and 10 mg/L vitamin B_1_; the mycelia grew well at pH 6.6 and 25 °C. The average biological efficiencies of fruiting bodies on wheat and rice as culture media were 1.880% and 2.115%, respectively; the polysaccharide contents of fruiting bodies on the two media were 6.635% and 9.264%, respectively, while the adenosine contents were 0.145% and 0.150%, respectively. This study provides a valuable reference for further artificial cultivation and utilization of *B. caledonica* by investigating its biological characteristics, cultivation conditions for artificial domestication, and polysaccharide and adenosine contents in cultivated fruiting bodies.

## 1. Introduction

*Cordyceps* sensu lato (hereafter referred to as *Cordyceps* s.l.) is a group of complex organisms consisting of entomogenous fungi and hosts. The infestation hosts for these entomogenous fungal complexes generally include many types of organic substances, such as insects, spiders, some fungi [1,2], plants [3], and rotifers [4], as well as inorganic substances, such as rocks, soil, and fresh water [5]. These entomogenous fungi are primarily distributed among nearly 200 genera within the Hypocreales order and belong to the Clavicipitaceae, Cordycipitaceae, Ophiocordycipitaceae, and Polycephalomycetaceae families [6]. Currently, there are approximately 1300 recorded species of *Cordyceps* s.l. worldwide [7,8]. However, only a few species form fruiting bodies. Among the numerous species of *Cordyceps* s.l. identified thus far, only *Ophiocordyceps sinensis* [9,10], *Cordyceps chanhua* [11], *Cordyceps militaris* [12], and *Tolypocladium guangdongense* [13] have been cultivated for commercial use.

In the natural world, there are many asexual and a few sexual species of *Cordyceps* s.l. belonging to *Beauveria* [14], *Metarhizium* [15,16], *Tilachlidium* [17,18], *Tolypocladium* [7], and others. *Beauveria* spp. are a class of entomogenous fungi that can parasitize 15 orders, 149 families, and more than 700 species of insects, including Lepidoptera, Coleoptera, Hymenoptera, and some tick mites [19]. According to the fungal classification system, *Beauveria* belongs to Ascomycota, Sordariomycetes, Hypocreales, and Cordycipitaceae [20]. Out of approximately 1300 species records of *Cordyceps* s.l., 81 records belong to *Beauveria* [8], among which China has 18 records of species, namely, *B. amorpha* [21], *B. araneola* [22], *B. baoshanensis* [23], *B. bassiana* [24], *B. brongniartii* [24], *B. caledonica* [25], *B. lii* [26], *B. majiangensis* [27], *B. malawiensis* [28], *B. medogensis* [29], *B. polyrhachicola* [30], *B. scarabaeidicola* [31], *B. sinensis* [32], *B. sobolifera* [33], *B. songmingensis* [30], *B. subscarabaeidicola* [30], *B. velata* [24], and *B. yunnanensis* [23]. As species of *Cordyceps* s.l., *Beauveria* spp. have characteristics such as a wide host range, strong pathogenicity, and strong selectivity [34]. Related studies on *Beauveria* have focused mainly on agricultural and forestry pest control [35]. Insecticidal preparations of *Beauveria* have been widely used in the control of agricultural and forestry pests, such as *Ostrinia furnacalis* [36] and *Frankliniella occidentalis* [37], which play important roles in promoting the development of agricultural and forestry industries as well as maintaining the stability of natural ecology. However, recent studies have shown that *Beauveria* insecticide may cause harm to the silkworm breeding industry [38], resulting in economic losses for farmers and, thus, limiting its application to some extent. Other studies have shown that *Bombyx batryticatus* is a coconstituted complex organism that is a traditional Chinese medicine formed after *Bombyx mori* is infected by *Beauveria bassiana* [39].

There has been no study on the artificial domestication and cultivation of fruiting bodies of *Beauveria*. This may be another area where *Beauveria* can be developed and utilized by humans in the future. The successful domestication and cultivation of *Beauveria* is highly important for the comprehensive development and utilization of *Cordyceps* s.l. resources [40]. A strain of *B. caledonica* collected from the wild was isolated and identified in this research, and its biological characteristics and artificial domestication cultivation were studied. Furthermore, the bioactive components of *B. caledonica* obtained from the fruiting bodies, such as polysaccharides, adenosine, and cordycepin, were studied, thus providing a reference for the comprehensive development and utilization of this fungus.

## 2. Materials and Methods

### 2.1. Materials

#### 2.1.1. Sample Collection

**Figure 1:**
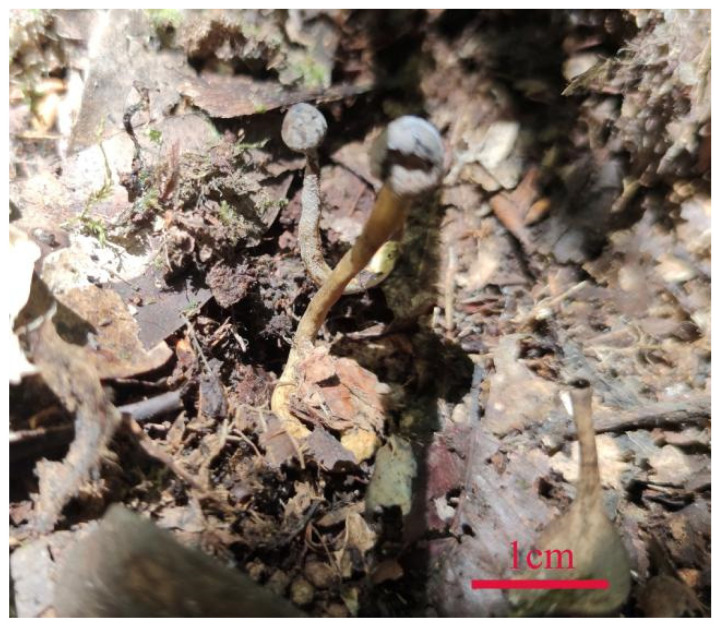
Wild fruiting bodies of *B. caledonica*.

Wild fruiting bodies of *B. caledonica* (Figure 1) were collected from Leigongshan National Nature Reserve in Leishan County, Qiandongnan Miao and Dong Autonomous Prefecture, Guizhou Province, China, at an altitude of 1912 m. Specimen number DCY 4974 was collected by Qunying Lin, Chuanhua Li, and Chunying Deng on 23 July 2022. Strains were obtained through tissue isolation using wild fruiting bodies as materials.

#### 2.1.2. Culture Medium

PDA medium (PDB medium without agar): 200 g of potato was peeled and boiled to extract the juice; 20 g of glucose and 20 g of agar were added and the volume was increased to 1 L with distilled water. Basal medium: 20 g of glucose, 10 g of peptone, 20 g of agar (liquid medium without agar), 1 g of MgSO_4_·7H_2_O, 1 g of KH_2_PO_4_, and 10 mg of vitamin B_1_ were mixed; the volume was increased to 1 L with distilled water. The above media were sterilized for 20 min at 121 °C.

Cultivation medium: In a 350 mL culture bottle containing 30 g of wheat/rice, 30 mL of nutrient solution was added, with 10 replications in each group, and the medium was sterilized for 40 min at 121 °C. The nutrient solutions used were as follows: 26.2 g of glycerol, 11.1 g of yeast powder, 1 g of MgSO_4_·7H_2_O, 1 g of KH_2_PO_4_, and 10 mg of vitamin B_1_ in 1 L of distilled water.

### 2.2. Methods

#### 2.2.1. Molecular Biological Identification of the Strain

The mycelia were collected and ground thoroughly in liquid nitrogen, and DNA was extracted using a fungal DNA extraction kit (Yisheng Biotechnology Shanghai Co., Ltd., Shanghai, China) and then stored at −20 °C until further use. PCR amplification was performed using the primers ITS4 (5′-TCCTCCGCTTATTGATATGC-3′) and ITS5 (5′-GGAAGTAAAAGTCGTAACAAGG-3′) (synthesized by Sangon Biotech (Shanghai) Co., Ltd., Shanghai, China). The reaction system included 1 μL of template DNA, 12.5 μL of 2×Taq PCR Master Mix (Vazyme, Nanjing, China), 1 μL of each primer, and 9.5 μL of ddH_2_O. The PCR amplification program was as follows: predenaturation at 94 °C for 4 min, followed by 30 cycles of denaturation at 94 °C for 30 s, annealing at 55 °C for 30 s, extension at 72 °C for 1 min, and a final extension at 72 °C for 10 min.

The amplified products were sequenced by Sangon Biotech (Shanghai) Co., Ltd., and the measured sequences were compared with the DNA database with the BLAST tool on the NCBI website. The MEGA-X neighbor-joining (NJ) method was used to construct a phylogenetic tree, the bootstrap method was used, and the bootstrap parameter was set to 1000 to establish a p-distance model, with the other parameters set to the defaults.

#### 2.2.2. Single-Factor Experiment

Temperature experiment: PDA medium was used to measure the growth rate of mycelia, and 20 mL of PDA medium was poured into Petri dishes. After inoculation, the cultures were incubated in a dark environment at a constant temperature of 15, 20, 23, 25, 28, or 30 °C. The biomass (dry weight) of the mycelia in the PDB medium was measured, and 100 mL of PDB medium was added to 250 mL flasks. After inoculation, the flasks were incubated at a constant temperature with shaking at 150 rpm in the dark. Each experiment was repeated four times.

Carbon source experiment: Basal medium (solid culture supplemented with 20 g/L agar powder) was selected; glucose, sucrose, maltose, fructose, glycerol, and soluble starch were used as carbon sources (20 g/L); and no carbon source was added as the control (CK). Peptone was used as the fixed nitrogen source (10 g/L), and, after inoculation, the fungi were cultured at 25 °C. Each experiment was repeated four times.

Nitrogen source experiment: Basal medium (solid culture supplemented with 20 g/L agar powder) was selected, yeast extract, peptone, soy peptone, beef extract, (NH_4_)_2_SO_4_, and silkworm pupae powder were used as nitrogen sources (10 g/L), and no nitrogen source was added as the control (CK). Glucose was used as the fixed carbon source (20 g/L), and, after inoculation, the fungi were cultured at 25 °C. Each experiment was repeated four times.

pH experiment: The media used were as follows: 20 g of glycerol, 10 g yeast extract, 1 g of MgSO_4_·7H_2_O, 1 g of KH_2_PO_4_, and 10 mg of vitamin B_1_ were added to 1 L of distilled water, and the media were sterilized for 20 min at 121 °C. The pH of the culture media was adjusted to 5, 6, 7, 8, or 9 using 1 M NaOH or 1 M HCl solution. Each experiment was repeated four times.

#### 2.2.3. RSM Experiment

The glycerol content (A), yeast extract content (B), and pH (C) were selected to optimize the mycelial growth rate and biomass according to the single-factor experimental results. The three-factor, three-level combined Box–Behnken method was used for the RSM experiment (Table 1).

#### 2.2.4. Determination of the Growth Rate and Biomass of Mycelia

The growth rates of the mycelia were determined on solid medium and the cross method (measuring the colony growth radius), and the mycelia were observed and marked at 5 d and 20 d after inoculation. The growth rates of the mycelia (mm/d) were calculated as follows: (the second marked radius length—the first marked radius length)/days between two markers.

The biomass of the mycelia was measured in liquid media. After 4 d of cultivation, the mycelia were collected, washed with ddH_2_O, filtered with filter paper, and dried at 55 °C to a constant weight, after which their dry weight was measured.

#### 2.2.5. Domestication Cultivation

Preparation of cultivated spawn: Liquid spawn were used as cultivated spawn after 4 d of cultivation.

Inoculation: After sterilization and cooling to room temperature, each sterilized cultivation medium was inoculated with 5 mL of cultivated spawn on an ultraclean bench.

Mushroom production management: After inoculation, the culture bottles were placed in a 25 °C incubator in the dark. When the medium was covered with mycelia, the medium was illuminated for 12 h (2000 lux) per day, and the other culture conditions were unchanged. Twenty days after the surface of the mycelia formed a bundle and then reached the shoulder of the bottle, the fruiting bodies were harvested.

#### 2.2.6. Polysaccharide Detection in Fruiting Bodies

Glucose solutions were prepared (0, 0.01, 0.02, 0.04, 0.06, 0.08, and 0.10 mg/mL). Fruiting body powder (0.05 g) was suspended in 1 mL of distilled water and then extracted in a 100 °C water bath for 2 h. After that, the solution was cooled to room temperature, the lost water was replenished, the mixture was centrifuged at 10,000× *g* for 10 min, and the supernatant was retained. One milliliter of distilled water was added to the precipitate, the above steps were repeated twice, and the supernatant was concentrated to 1 mL (the supernatant containing the suspended spore powder was filtered with a 0.22 μm cellulose filter membrane to remove impurities). Then, 0.2 mL of the supernatant was suspended in 0.8 mL of anhydrous ethanol, placed at 4 °C for 12 h, and centrifuged at 10,000× *g* for 10 min. The supernatant was discarded, and 1 mL of distilled water was added for precipitation. Then, 0.2 mL of sample solution (20-fold diluted) was added to 0.1 mL of 5% phenol solution and 0.5 mL of concentrated sulfuric acid. After vortexing, the mixture was placed in a 90 °C water bath for 20 min. After cooling with running water, 0.2 mL of solution was transferred to an enzyme-linked immunosorbent assay (ELISA) plate, and the absorbance was measured at 490 nm [41].

#### 2.2.7. Adenosine and Cordycepin Detection in Fruiting Bodies

Chromatographic conditions: A Sunfire C18 column (4.6 mm × 250 mm, 5 μm) was used as the analytical column. Methanol and water (15:85, *v*/*v*) were used as the mobile phase, with a flow rate of 1 mL/min, a detection wavelength of 260 nm at 25 °C, and an injection volume of 10 μL [42].

Standard curve experiments: A mixed standard solution of adenosine and cordycepin (0, 2.5, 5, 10, 20, 40, 60, 80, and 100 μg/mL) was prepared to obtain the calibration curves, and the relative correction factors were calculated. Standard curves of the investigated compounds were established by plotting the peak areas (Y) versus the concentration of standard compound (X, μg/mL).

Sample preparation: Powdered fruiting bodies (0.15 g) were suspended in 10 mL of ultrapure water, and after sonication for 1 h, the solution was centrifuged at 7000× *g* for 20 min. The supernatant was filtered with a filter membrane (0.22 μm cellulose filter, Millipore, Shanghai, China) and then analyzed according to the chromatographic conditions, with three replicates for each sample.

Recovery detection: The recovery experiment was performed by adding an equal volume of 20 μg/mL mixed standard solution to a known concentration of sample solution of fruiting bodies. Then, these samples were prepared and analyzed through chromatography, and the recovery rate was calculated.

### 2.3. Data Analysis

Data analysis and significant differences were assessed by using Microsoft Excel and SPSS 25.0 software. Visual graphs were generated with Origin 2018, and the RSM model and standard deviations were generated with Design-Expert10 software.

## 3. Results

### 3.1. Molecular Analysis

**Figure 2:**
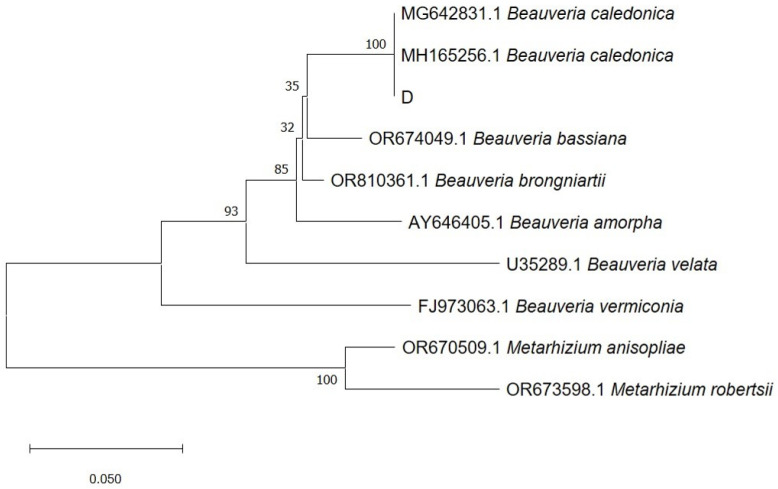
Phylogenetic tree constructed from the ITS sequences of *B. caledonica* and two other species of *Metarhizium.* Scale bar indicates 0.050 sequence divergence substitutions per nucleotide position.

The ITS amplification sequence was cleaved and spliced using ContigExpress alignment program in Vector NTI version 9.0 software (Invitrogen corporation, Carlsbad, CA, USA), and a 525 bp DNA fragment was obtained (accession number: SUB14585645), which shared more than 99% homology with three fragments of *B. caledonica* in the GenBank database (accession numbers: MG642831, MH165256, and MT180426). The constructed phylogenetic tree was divided into two main branches, one composed of *M. anisopliae* and *M. robertsii* and the other consisting of sample D, *B. caledonica* (MG642831, MH165256), *B. bassiana* (OR674049), *B. brunniarteii* (OR810361), *B. amorpha* (AY646405), *B. velata* (U35289), and *B. vermiconia* (FJ973063). The studied sample clustered on the same branch as the two *B. caledonica*. The sample was, therefore, identified as *B. caledonica* (Figure 2). The identification was done with one single molecular marker and may be other molecular markers such as TEF1-α are needed to confirm the molecular identity of the isolate at the species level further.

### 3.2. Temperature Experiment

**Figure 3:**
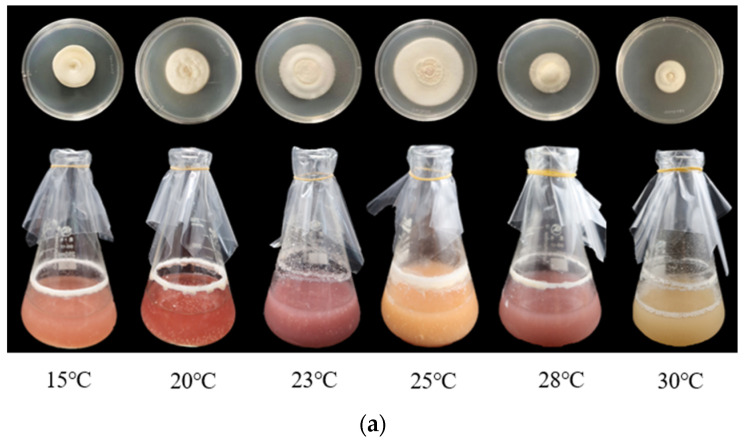

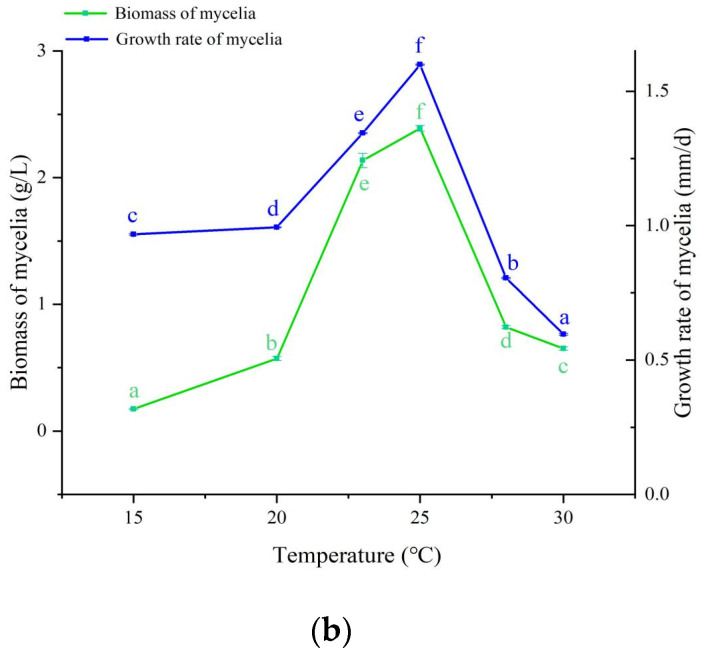
Effects of temperature on the mycelial growth of *B. caledonica.* (**a**) The mycelia of *B. caledonica* grew in PDA and PDB media at different temperatures. (**b**) The biomass of *B. caledonica* mycelia at different temperatures. Data are shown as the mean ± standard deviation (n = 4). The lowercase letters indicate a significant difference at the level of 0.05 (*p* < 0.05).

The mycelia of *B. caledonica* could grow in PDA and PDB media at different temperatures (15, 20, 23, 25, 28, and 30 °C), and the optimal temperature was 25 °C for both cultivation methods (the fastest mycelia growth rate and the greatest mycelia biomass) (Figure 3a). Mycelia were cultured under solid culture conditions, and the growth rates were sequentially slower in this order: 25, 23, 20, 15, 28, and 30 °C. The biomass of the mycelia also decreased sequentially in the following order: 25, 23, 28, 30, 20, and 15 °C under liquid culture conditions (Figure 3b).

### 3.3. Carbon Source Experiment

**Figure 4:**
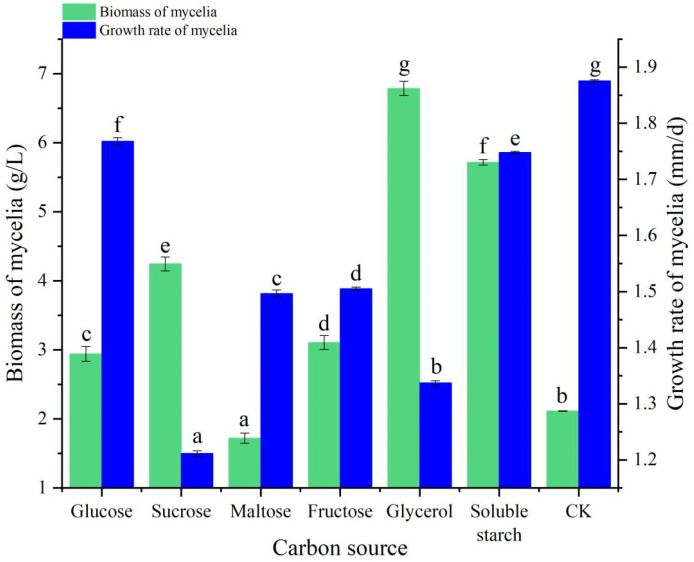
Effects of different carbon sources on the mycelial growth of *B. caledonica.* Data are shown as the mean ± standard deviation (n = 4). Bar charts with different letters are significantly different (*p* < 0.05).

The mycelia of *B. caledonica* could grow on solid and liquid media with different carbon sources (glucose, sucrose, maltose, fructose, glycerol, and soluble starch) and on the control (CK), but there were significant differences in mycelial growth among these conditions. The growth rates of mycelia on solid media sequentially decreased in this order: control, glucose, soluble starch, fructose, maltose, glycerol, and sucrose (Figure 4). The colonies were sparse when glucose, maltose, fructose, and soluble starch were used as carbon sources, and the colonies in the control group were also sparse. However, the colonies were dense, and the mycelial surface was smooth and pure white when sucrose and glycerol were used as carbon sources. The biomass of mycelia in liquid media decreased sequentially in this order: glycerol, soluble starch, sucrose, fructose, glucose, no carbon source, and maltose. In general, the optimal carbon source for both cultivation methods was glycerol (Figure 4).

### 3.4. Nitrogen Source Experiment

**Figure 5:**
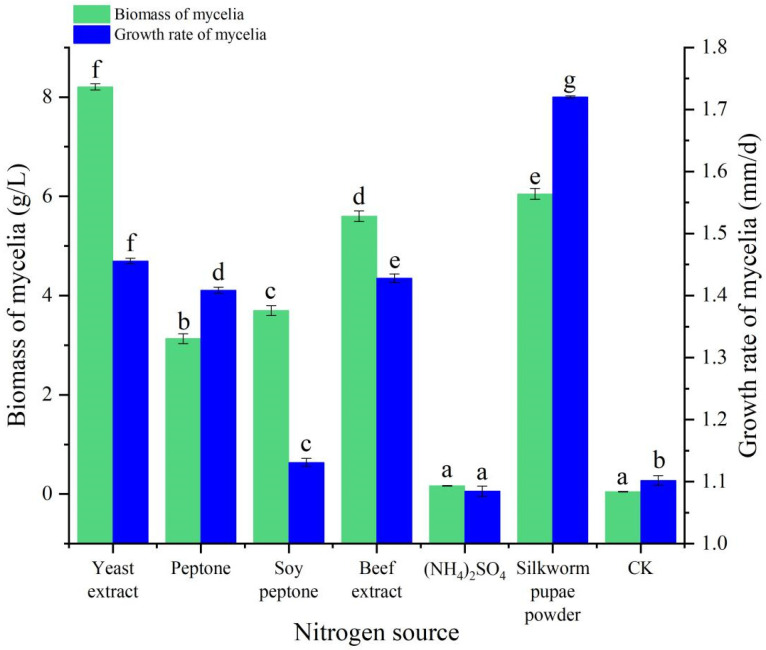
Effects of different nitrogen sources on the mycelial growth of *B. caledonica.* Data are shown as the mean ± standard deviation (n = 4). Bar charts with different letters are significantly different (*p* < 0.05).

The mycelia of *B. caledonica* could grow on solid and liquid media with different nitrogen sources (yeast extract, peptone, soy peptone, beef extract, (NH_4_)_2_SO_4_, and silkworm pupae powder) and on the control, but there were significant differences in mycelial growth. The growth rate of the mycelia decreased sequentially in the following order: silkworm pupae powder, yeast extract, beef extract, peptone, soy peptone, control, and (NH_4_)_2_SO_4_ (Figure 5). The colonies were sparse when beef extract, (NH_4_)_2_SO_4_, and silkworm pupae powder were used as nitrogen sources, and the colonies in the control group were also sparse. However, the colonies were dense when yeast extract, peptone, and soy peptone were used as nitrogen sources, and the colonies were whiter and denser when yeast extract was used as a nitrogen source than when other nitrogen sources were used. In liquid culture, the biomass of the mycelia decreased sequentially in the following order: yeast extract, silkworm pupae powder, beef extract, soy peptone, peptone, (NH_4_)_2_SO_4_, and control, and there was no significant difference between (NH_4_)_2_SO_4_ and the control (Figure 5). In general, the optimal nitrogen source for both cultivation methods was yeast extract.

### 3.5. pH Experiment

**Figure 6:**
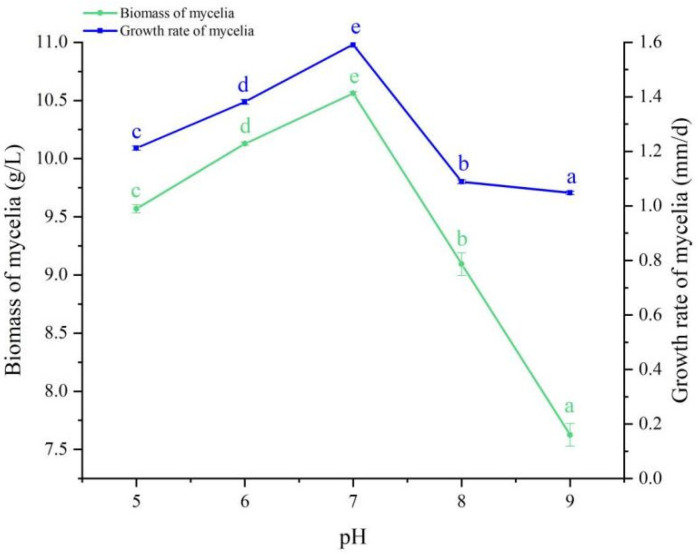
Effects of pH on the mycelial growth of *B. caledonica.* Data are shown as the mean ± standard deviation (n = 4). The lowercase letters indicate a significant difference at the level of 0.05 (*p* < 0.05).

The mycelia of *B. caledonica* could grow on solid and liquid media with different pH values (pH 5, 6, 7, 8, and 9). The growth rate of mycelia decreased sequentially in the following order: pH 7, 6, 5, 8, and 9. The biomass of the mycelia in liquid culture decreased sequentially in the following order: pH 7, 6, 5, 8, and 9. The two cultivation methods had the same change trend with respect to pH; therefore, the optimal pH was 7 for both liquid and solid cultivation (Figure 6).

### 3.6. RSM Experiment

#### 3.6.1. Statistical Analysis Using RSM

To optimize mycelial growth, the glycerol content, yeast extract content, and pH were selected as independent variables in the single-factor experiments. The growth rate and biomass were optimized using the RSM approach combined with the Box–Behnken method. The complete experimental design is shown in Table 2, consisting of 17 runs with independent variables at three variable levels.

A total of 17 runs were performed to optimize each parameter in the Box–Behnken design. The regression equation of response can be predicted by examining the independent and dependent variables. The mathematical model for predicting the response *Y* (growth rate) of *B. caledonica* can be represented by the following quadratic formula:

*Y*_growthrate_

= −33.31694 + 0.056245A + 0.16851B + 10.26720C + 0.0012875AB − 0.000575AC + 0.00303BC − 0.00133862A^2^ − 0.0099505B^2^ − 0.78645C^2^

The mathematical model for predicting the response *Y* (biomass) of *B. caledonica* can be represented by the following quadratic formula:

Y_biomass_

= 178.85926 + 0.27031A + 1.05365B + 55.19357C + 0.0068025AB + 0.009685AC + 0.01876BC − 0.0083162A^2^ − 0.061738B^2^ − 4.24398C^2^

The models showed that three factors had a nonlinear relationship with the growth rate and biomass of mycelia, and there was an interaction effect among the independent variables.

#### 3.6.2. Analysis of Variance (ANOVA) of Regression Equations

A good quadratic model for the growth rate of *B. caledonica* was obtained. According to the ANOVA, the model was extremely significant (*p* < 0.0001), and the degree of fit was good, which could reflect the relationships between the mycelial growth rate of *B. caledonica* and the independent variables (Table 3). The difference in lack of fit was not significant (*p* > 0.05), indicating that the model fit well throughout the entire regression area. The resulting R^2^ = 0.9888 and *R*_Adj_^2^ = 0.9743 of the regression model showed that three independent variables in the test had an impact on the response value of 98.88%, and only 2.57% of the total variation could not be fitted by the model. A C.V. of 2.19% indicates the high reliability and accuracy of the test, and this mathematical model can be used to predict the experimental results. The regression coefficients A, B, and C, interaction coefficient AB, and quadratic coefficients A^2^, B^2^, and C^2^ were all significant (*p* < 0.01), indicating that glycerol content, yeast extract content, and pH were significantly correlated with the growth rate of the mycelia. The glycerol content was shown to be the major factor impacting the growth rate of the mycelia, followed by the yeast extract content and pH, according to a comparison of F (A) = 94.12 > F (B) = 37.53 > F (C) = 6.49 (Table 3).

According to the ANOVA of the biomass, the quadratic model was extremely significant (*p* < 0.0001), and the degree of fitting was good, which could reflect the relationship between the mycelial biomass of *B. caledonica* and the independent variables (Table 4). The difference in lack of fit was not significant (*p* > 0.05), indicating that the model fit well throughout the entire regression area. The resulting *R*^2^ = 0.9952 and *R*_Adj_^2^ = 0.9890 of the regression model showed that three independent variables in the test had an impact on the response value of 99.52%, and only 1.10% of the total variation could not be fitted by the model. The C.V. of 1.43% indicates the high reliability and accuracy of the test, and the mathematical model can be used to predict the experimental results. The regression coefficients A, B, and C, interaction coefficient AB, and quadratic coefficients A^2^, B^2^, and C^2^ were all significant (*p* < 0.01), indicating that glycerol content, yeast extract content, and pH were significantly correlated with the growth rate of the mycelia. The glycerol content was shown to be the major factor impacting the growth rate of the mycelia, followed by the yeast extract content and pH, according to a comparison of F (A) = 216.28 > F (B) = 67.83 > F (C) = 18.65 (Table 4).

#### 3.6.3. RSM Analysis of the Interaction of Factors

**Figure 7:**
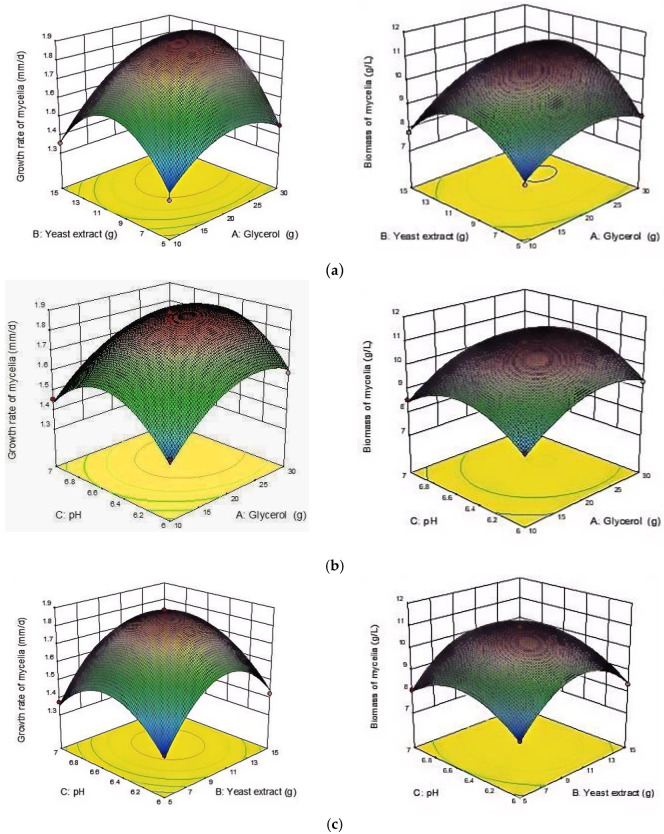
The independent variable response surface 3D maps of *B. caledonica* mycelia under the two culture methods. (**a**) AB response surface map; (**b**) AC response surface map; (**c**) BC response surface map. A: glycerol content; B: yeast extract content; C: pH.

The interactions between the variables were examined by a three-dimensional (3D) response surface plot through Design Expert 10 software. To optimize the growth rate and biomass, graphs were created to show the correlation between the response and the level of the variable, as well as the interactions among the variables. The steepness of the surface in this 3D map can indicate the extent to which the three independent variables affect the change in the growth rate and biomass of the mycelia. According to Figure 7, it was observed that when the glycerol content ranged from 25 to 30 g and the yeast extract content ranged from 9 to 15 g, the mycelial growth rate and biomass were greater (Figure 7a). Similarly, when the glycerol content was within the range of 25–30 g and the pH was maintained at 6.4–6.8, there was also an increase in mycelial growth rate and biomass (Figure 7b). Additionally, when the yeast extract content fell between 9 and 15 g and the pH remained at 6.4–6.8, there was a similar enhancement in mycelial growth rate and biomass (Figure 7c). The steepness and corresponding contour plots displayed significant changes in Figure 7a, indicating that both glycerol content and yeast extract content had substantial effects on mycelial growth rate and biomass. However, the interactions between glycerol content and pH as well as yeast extract content and pH were found to be insignificant in Figure 7b,c. These findings are consistent with the ANOVA results of the response surfaces presented in Table 3 and Table 4. Combined with the quadratic regression equation in this study, the results showed that the glycerol content and yeast extract content had an extremely significant interaction effect (*p* < 0.01), and the response value showed a parabolic trend (Figure 7a). Therefore, the regression equation had a maximum value.

#### 3.6.4. RSM Optimization Validation

According to the analysis by the Numerical Optimization module of Design-Expert 10.0.1 software, the optimal theoretical formula for the growth rate of solid cultured mycelia was 25.783 g glycerol, 10.919 g yeast extract, and pH 6.551. The theoretical mycelial growth rate under the optimal conditions was 1.889 mm/d. However, for the convenience of operation, the following formula was used: 25.8 g of glycerol, 10.9 g of yeast extract, pH 6.5, 1 g of MgSO_4_·7H_2_O, 1 g of KH_2_PO_4_, 10 mg of vitamin B_1_, 20 g of agar, and 1 L of distilled water, with four replicates. The final experimental mycelial growth rate was 1.890 mm/d, which was approximately the expected value.

The theoretical formula for the maximum biomass of liquid-cultured mycelia yielded optimal conditions of 26.171 g glycerol, 11.076 g yeast extract, and pH 6.613. Under these conditions, the theoretical mycelial biomass calculated by the formula was 11.036 g/L. Using the actual operating formula, these numbers were 26.2 g glycerol, 11.1 g yeast extract, pH 6.6, 1 g MgSO_4_·7H_2_O, 1 g KH_2_PO_4_, 10 mg vitamin B_1_, and 1 L distilled water, and this medium was tested with four replicates. The final experimental mycelial biomass was 11.020 g/L, which was approximately the expected value. In general, the quadratic multiple regression equation model had a certain degree of practical predictability.

### 3.7. Domestication Cultivation Experiment

**Figure 8:**
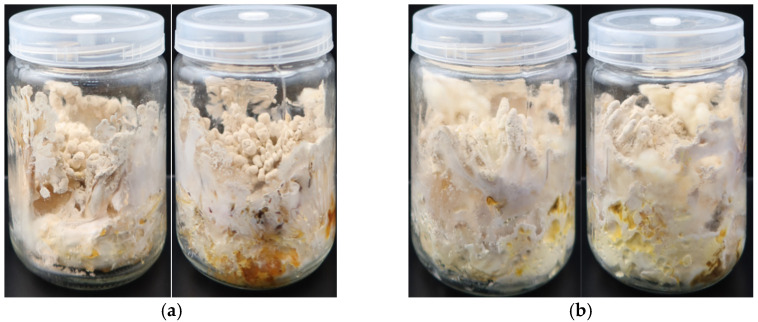
Domestication cultivation of *B. caledonica.* (**a**) Wheat cultivation medium; (**b**) rice cultivation medium.

Figure 8 shows the differences in the domestication and cultivation of *B. caledonica* between wheat cultivation medium (Figure 8a) and rice cultivation medium (Figure 8b). There was a significant difference in the growth rate of *B. caledonica* mycelia between the two different cultivation media. *B. caledonica* could grow normally in both cultivation media, and the time for the mycelia to completely fill the cultivation bottle was approximately 10–11 d in the wheat cultivation medium, whereas it took 13–15 d in the rice cultivation medium. After the mycelia were fully grown, the bottles were cultured under light for 12 h/d. Approximately 20 d later, the mycelia had adhered to the wall of the bottle and had grown to approximately two-thirds of the height of the bottle. However, no primordia or fruiting bodies formed on the surface of the mycelia at this time. After approximately 20 d of further culture, fruiting bodies formed from the surface of the mycelia in the middle of the cultivation medium, and aleuriospores attached to the surface of the fruiting bodies. The fruiting bodies could be harvested after 15 d of further culture when they had grown to the height of the adherent mycelia. Therefore, the entire cultivation cycle lasted approximately 65 d.

The average yields of fruiting bodies produced by the wheat cultivation medium and rice cultivation medium were 0.470 g and 0.529 g, respectively (Table 5). There was no obvious difference in the morphology of the fruiting bodies produced by the two cultivation media, and the heads of the fruiting bodies were swollen and elliptical, branching or not branching, with villi on the surface and some spore powder attached. The stem of the fruiting bodies was often branched, with villi on the surface. The single fruiting bodies had a soft texture and a gap inside (Figure 8a,b).

### 3.8. Polysaccharide Content of Fruiting Bodies

The glucose standard curve equation was Y = 8.8179x + 0.0492, and the correlation coefficient *R*^2^ = 0.999.

$$\mathrm{Polysaccharide}\;\mathrm{content}\;(\%)=\frac{\left(A-0.0492\right)\times 100\times 100}{W\times 8.8179}$$

*A*: absorbance value; *W*: weight of fruiting bodies (mg).

The polysaccharide contents of the fruiting bodies produced by the wheat cultivation medium and rice cultivation medium were 6.635 ± 0.059% and 9.264 ± 0.053%, respectively. The polysaccharide content of the fruiting bodies produced by the rice cultivation medium was greater than that produced by the wheat cultivation medium.

### 3.9. Adenosine and Cordycepin Contents in Fruiting Bodies

**Figure 9:**
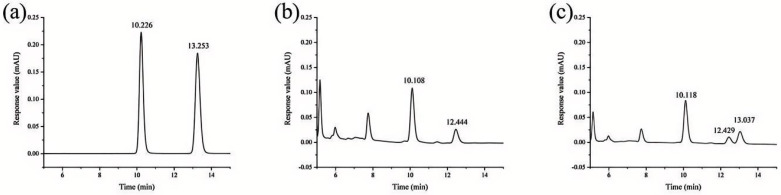
HPLC chromatogram of the adenosine and cordycepin in *B. caledonica*. (**a**) A 100 μg/mL adenosine and cordycepin standard solution; (**b**) mixed sample liquid chromatogram; (**c**) addition of adenosine and cordycepin standard solution to the mixed sample solution. The chromatographic peak with a retention time of 10.226 min was attributed to adenosine, and the peak with a retention time of 13.253 min was attributed to cordycepin.

The chromatographic peaks of the adenosine and cordycepin standards were obvious and symmetrical, with no miscellaneous peak interference, indicating that the purity of the mixed standard solution was good (Figure 9). As shown in Figure 9a, the chromatographic peak with a retention time of 10.226 min was attributed to adenosine, and the peak with a retention time of 13.253 min was attributed to cordycepin. Two peaks with high response values appeared at 10-14 min in the mixed sample solution, with retention times of 10.108 and 12.444 min, respectively (Figure 9b). Three peaks with high response values appeared at 10–14 min, as shown in Figure 9c. Compared with Figure 9a,b, the chromatographic peak with a retention time of 10.118 min was adenosine, and the peak with a retention time of 13.037 min was cordycepin. Adenosine was detected in the fruiting bodies of *B. caledonica*, but cordycepin was not detected.

The average recovery rate of adenosine was 98.175%, and the RSD was 0.955%. The results showed that the standard compound was recovered well and could be used to detect the adenosine content of fruiting bodies in *Cordyceps* in this study (Table 6). The regression equation for the adenosine standard curve was y = 32617x + 3788.5, and the correlation coefficient *R*^2^ = 0.9994. According to the standard curve equation, the adenosine content of fruiting bodies produced in wheat medium was 0.145 ± 0.001%, and the adenosine content of fruiting bodies produced in rice medium was 0.150 ± 0.001%.

## 4. Discussion

As a rare species of *Cordyceps* s.l., *B. caledonica* has only been studied in field investigations for its antibacterial activities and insect toxicity. The biological characteristics, artificial domestication, and cultivation of this fungus have not been reported and need to be further studied [43,44]. In this research, the ITS4/ITS5 primers were used to amplify the ITS sequence of the wild fruiting bodies, which was identified by homologous and phylogenetic analysis. It was found that the wild strain was *B. caledonica* based on molecular identification. Because of high degree of interspecific variability, conserved primer sites and multiple copy nature in the genome, the potential barcode ITS molecular marker for most of fungi was used for the identification. Other molecular markers such as TEF1-α may be use as secondary barcode to confirm the molecular identity of wild strain, which may be more effective for species level identification [45]. The isolated strain was further used as the study object to explore the optimal mycelial culture conditions and domestication cultivation of fruiting bodies. We found that different liquid media exhibited different colors when used to cultivate the mycelia, which may be related to the pigments secreted during the *B. caledonica* growth process. According to Jian et al., the yellow pigment of *C. militaris* could be widely used in the food industry because of its beneficial physiological properties, such as anti-inflammatory and antioxidant effects [46]. Therefore, subsequent research should be performed on the biological pigments of *B. caledonica,* which has great potential for pigment production.

Fruiting bodies often consist of conidiophores with dense, sticky or soft, upright or curved, branched or unbranched morphologies and are clustered into sporulation structures [47]. In our previous study, we found that when rice or wheat were used as cultivation media under humid (air relative humidity 90 ± 5%), ventilated (opening the bottle cap), and light conditions (12 h/d), the mycelia could completely fill the whole culture bottle, but they could not form fruiting bodies even after approximately 50 days of cultivation. Our further study showed that the fruiting bodies only grew well under light conditions (12 h/d) for mushroom production management, excluding humidification and ventilation conditions. Under the same cultivation conditions, the fruiting bodies produced by *B. caledonica* were different from those produced by *Cordyceps tenuipes* and *Polycephalomyces ramosus* and had a soft texture, light weight, and low yield [48,49]. The domestication and cultivation of *B. caledonica* indicated that artificial regulation to produce a dry and low-oxygen culture environment is an effective way to promote the transformation of some difficult to domesticate *Cordyceps* from asexual propagation to fruiting bodies.

The fruiting bodies of *Cordyceps* have medicinal value. *Cordyceps* has a high content of polysaccharide, which is an important bioactive substance that has been widely used in health and medical products because of its physiological functions, such as improving immunity, and antiaging, anticancer, and antioxidative effects [50]. In this study, the polysaccharide content of fruiting bodies produced by *B. caledonica* was greater than that produced by *P. ramosus* [49]. Therefore, subsequent research should be performed on the polysaccharide activity and function of *B. caledonica*. In addition, the minimum adenosine content of fruiting bodies in this study was 0.145 ± 0.001%, which was much greater than the standard of the pharmacopoeia. The adenosine content in *Cordyceps* is not less than 0.010% according to the Pharmacopoeia of the People’s Republic of China (2020 edition), suggesting that the fruiting bodies of *B. caledonica* in this study may have high medicinal value. However, cordycepin was not detected in this study. This study provides a reference for the artificial cultivation and utilization of *B. caledonica*.

## Figures and Tables

**Table 1:** Values of the factors and the corresponding levels in the RSM.

Independent Variables	Symbol	Levels		
		−1	0	1
Glycerol content (g/L)	A	10	20	30
Yeast extract content (g/L)	B	5	10	15
pH	C	6.0	6.5	7.0

**Table 2:** Experimental matrix with results for response surface methodology.

Run	Coded Variable Levels			Response Value	
	A Glycerol Content(g)	B Yeast Extract Content(g)	C pH	Growth Rate of Mycelia (mm/d)	Biomass of Mycelia (g/L)
1	10	5	6.5	1.3006	7.6249
2	30	5	6.5	1.4537	8.5149
3	10	15	6.5	1.3555	7.799
4	30	15	6.5	1.7661	10.0495
5	10	10	6	1.3933	8.2417
6	30	10	6	1.5907	9.3202
7	10	10	7	1.4573	8.542
8	30	10	7	1.6432	9.8142
9	20	5	6	1.3231	7.7684
10	20	15	6	1.4233	8.3579
11	20	5	7	1.374	8.0837
12	20	15	7	1.5045	8.8608
13	20	10	6.5	1.8884	10.8245
14	20	10	6.5	1.844	10.7834
15	20	10	6.5	1.8359	10.9635
16	20	10	6.5	1.8537	10.8246
17	20	10	6.5	1.836	10.9647

**Table 3:** Significance test and ANOVA of the regression equation (growth rate as the dependent variable).

Source	Sum of Squares	Df	Mean Square	*F* Value	*p*-Value	Significance
Model	0.73	9	0.082	68.49	<0.0001	**
A	0.11	1	0.11	94.12	<0.0001	**
B	0.045	1	0.045	37.53	0.0005	**
C	7.725 × 10^−3^	1	7.725 × 10^−3^	6.49	0.0383	*
AB	0.017	1	0.017	13.92	0.0074	**
AC	3.306 × 10^−5^	1	3.306 × 10^−5^	0.028	0.8724	
BC	2.295 × 10^−4^	1	2.295 × 10^−4^	0.19	0.6739	
A^2^	0.075	1	0.075	63.35	<0.0001	**
B^2^	0.26	1	0.26	218.77	<0.0001	**
C^2^	0.16	1	0.16	136.66	<0.0001	**
Residual	8.337 × 10^−3^	7	1.191 × 10^−3^			
Lack of Fit	6.431 × 10^−3^	3	2.144 × 10^−3^	4.50	0.0902	
Pure Error	1.906 × 10^−3^	4	4.766 × 10^−4^			
Cor Total	0.74	16				
*R*^2^ = 0.9888 *R_Adj_*^2^ = 0.9743 C.V./% = 2.19						

Note: *: significant (*p* < 0.05); **: highly significant (*p* < 0.01).

**Table 4:** Significance test and ANOVA of the regression equation (with biomass as the dependent variable).

Source	Sum of Squares	Df	Mean Square	*F* Value	*p*-Value	Significance
Model	25.33	9	2.81	161.52	<0.0001	**
A	3.77	1	3.77	216.28	<0.0001	**
B	1.18	1	1.18	67.83	<0.0001	**
C	0.33	1	0.33	18.65	0.0035	**
AB	0.46	1	0.46	26.55	0.0013	**
AC	9.38 × 10^−3^	1	9.38 × 10^−3^	0.54	0.4870	
BC	8.798 × 10^−3^	1	8.798 × 10^−3^	0.5	0.5004	
A^2^	2.91	1	2.91	167.09	<0.0001	**
B^2^	10.03	1	10.03	575.55	<0.0001	**
C^2^	4.74	1	4.74	271.98	<0.0001	**
Residual	0.12	7	0.017			
Lack of Fit	0.093	3	0.031	4.21	0.0992	
Pure Error	0.029	4	7.33 × 10^−3^			
Cor Total	25.46	16				
*R*^2^ = 0.9952 *R_Adj_*^2^ = 0.9890 C.V./% = 1.43						

Note: **: highly significant (*p* < 0.01).

**Table 5:** Domestication cultivation of *B. caledonica*.

Cultivation Medium	Average Yield (g)	Dry/Wet Weight Ratio	Biological Efficiency (%)
Wheat	0.470 ± 0.047	0.403 ± 0.035	1.880 ± 0.188
Rice	0.529 ± 0.043	0.451 ± 0.034	2.115 ± 0.172

**Table 6:** Standard recovery detection of the adenosine content in *B. caledonica*.

Ingredient	Sample Concentration (μg/mL)	Standard Concentration (μg/mL)	Detect the Concentration (μg/mL)	Concentration Should be Measured (μg/mL)	Recovery (%)	Average Recovery (%)	RSD (%)
Adenosine	32.076	20	26.869	26.038	96.907	98.175	0.955
	32.108		26.738	26.054	97.441		
	32.112		26.397	26.056	98.708		
	32.078		26.331	26.039	98.893		
	32.096		26.331	26.048	98.926		

## Data Availability

The original contributions presented in the study are included in the article, further inquiries can be directed to the corresponding authors.
